# Targeted near infrared hyperthermia combined with immune stimulation for optimized therapeutic efficacy in thyroid cancer treatment

**DOI:** 10.18632/oncotarget.6901

**Published:** 2016-01-12

**Authors:** Le Zhou, Mengchao Zhang, Qingfeng Fu, Jingting Li, Hui Sun

**Affiliations:** ^1^ Department of Thyroid Surgery, China-Japan Union Hospital, Jilin University, Jilin Provincial Key Laboratory of Surgical Translational Medicine, Changchun 130033, China; ^2^ Radiology Department, China-Japan Union Hospital, Jilin University, Changchun 130033, China

**Keywords:** IR820, amino-glucose, near infrared hyperthermia, tumor targeting, heat shock protein 70

## Abstract

Treatment of thyroid cancer has incurred much focus because of its high prevalency. As a new strategy treating thyroid cancer, hyperthermia takes several advantages compared with surgery or chemotherapy, including minimal invasion, low systematic toxicity and the ability to enhance the immunogenicity of cancer cells with the expression Hsp70 which serves as Toll-like receptors-4 (TLR-4 agonist). However, Hsp70 as a molecular chaperone can protect cells from heat induced apoptosis and therefore compromise the tumor killing effect of hyperthermia. In this study, to solve this problem, a combined hyperthermia therapy was employed to treat thyroid cancer. We prepared a probe with the tumor targeting agent AG to monitor thyroid tumor issue and generate heat to kill tumor cells *in vivo*. At the same time Quercetin (inhibitor of HSP70) and lipopolysaccharide (LPS) (agonist of TLR-4) were used for the combined hyperthermia therapy. The results showed that compared with free IR820, AG modification facilitated much enhanced cellular uptake and greatly pronounced tumor targeting ability. The combined therapy exhibited the most remarkable tumor inhibition compared with the single treatments both *in vitro* and *in vivo*. These findings verified that the new therapeutic combination could significantly improve the effect of hyperthermia and shed light on a novel clinical strategy in thyroid cancer treatment.

## INTRODUCTION

Thyroid cancer is the most prevalent endocrine malignancy with increasing incidence through the past decade [1]. There are mainly four types of thyroid carcinoma according to histopathological classification, namely follicular thyroid carcinoma (FTC), papillary thyroid carcinoma (PTC), medullary thyroid carcinoma (MTC) and anaplastic thyroid carcinoma (ATC), among which PTC is predominant accounting for 75–85% of all thyroid cancer cases [2]. For most thyroid cancer patients with PTC and FTC, a favorable therapeutic effect can be achieved through thyroidectomy surgery and radioiodine ablation with the general survival rate of 85% at 10 years [3]. Despite excellent prognosis, management of the disease is complicated mainly by the high rate of local recurrence (20% patients) and distant metastasis (10% at 10 years) [4–5]. Currently, treatment options for thyroid cancer patients mainly include surgical removal and radioactive iodine therapy. However, entire tumor removal is difficult to achieve by surgery and postoperative complications are common among patients, including hypocalcemia (20%–30%) and laryngeal nerve injury (5%–11%) [6]. ^131^I radioactive therapy is largely limited by patient's health status and treatment tolerability. In addition, the side effects are very common, such as salivary gland dysfunction (> 40%), abnormally dry eyes (25%) and transient fertility reduction (20%) [7]. Therefore, new and optimized therapeutic strategies are needed in thyroid cancer therapy.

In recent years, hyperthermia has emerged as a promising modality for cancer treatment using near-infrared (NIR) dye for both heat generation and tumor imaging. Taking advantage of the deep tissue penetration property of NIR light, NIR dyes have gained broad application in optical imaging, photodynamic therapy and hyperthermia. For example, under exposure of NIR light of appropriate wavelength, these dyes will generate heat and kill cancer cells. Since ICG (indocyanine green) was approved by US Food and Drug administration (FDA) as a diagnostic aid, intense efforts has been invested to use such NIR dyes as *in vivo* imaging agents and heat generator for light controlled release [8–10]. However, ICG suffers from several limitations such as weak stability and short plasma residence time of about 3.5 minutes [11]. Recently, IR820, a commercially available NIR dye, has been used in various theragnostic platforms due to its better applicability. Fernandez-Fernandez A et al. [12] compared the heat generation and imaging properties of ICG and IR820, showing that IR820 provides nearly doubled degradation half-time, enhanced stability and prolonged image collection time with similar heat generation property to ICG. At a low concentration, IR820 is able to produce sufficient fluorescence for imaging and enough heat for hyperthermia under exposure of NIR light. A further study [13] reported the conjugation of IR820 with chitosan for optical imaging and hyperthermic inhibition of different cancer cell lines. Jun Qian et al. [14] used IR820 dopped organically modified silica for mice brain imaging and photodynamic therapy. Moreover, IR820 has also been used for imaging of injured tissues as reported by Suresh I Prajapati et al. [15], serving as a useful and inexpensive contrast agent. However, NIR dyes including IR820 suffer nonspecific bio distribution *in vivo*, and complete tumor ablation by hyperthermia alone is difficult [16]. Therefore, it is urgently needed to perfect NIR dye with suitable tumor targeting agent to facilitate tumor specific accumulation.

Amino-glucose (AG), a glucose analog, has been widely exploited in tumor targeting for its unique chemical properties [17–18]. After recognized by glucose transporter 1 (GLUT1) on cell membrane, AG is transported into cells and phosphorylated into 2-deoxyglucose-6-phosphate and remains in the phosphorylated state, leading to intracellular retention. This is because the absence of hydroxyl group in position 2 inhibits isomerization and thus precludes further metabolism of AG [19]. Such an advantage has been employed by many researchers to develop tumor-targeting probes and therapeutic agents with AG. For example, Zhang et al. developed Pyro-AG with pyropheophorbide for targeted NIR imaging and photodynamic treatment [20]. Jing Guo et al. armed different NIR dyes with AG to compare their imaging properties and *in vivo* clearance in tumor bearing nude mice, and further verified the role of GLUT1 expression in determining the targeting ability of AG [21]. Therefore, AG as a kind of small molecule compound which is easily uptaken by cancer cells is an ideal tumor targeting agent for *in vivo* imaging and treatment.

One acknowledged complication in hyperthermia therapy is the induction of heat shock proteins (HSPs) that may compromise the therapeutic efficiency by exerting cytoprotective and antiapoptotic effects, including preventing protein inactivation and blocking apoptotic signaling [22–23]. Previous studies have confirmed the protective role of Hsp70 in both cellular and animal levels and different strategies were developed using Hsp70 inhibition to assist hyperthermia treatment [24–25]. For example, J. A. Barnes et al. revealed that Hsp70 can promote the proliferation of MCF-7 breast tumor cells and protect cells against cytotoxic effects of hyperthermia [26]. Wei Yang et al. combined liposomal Quercetin, a well-known inhibitor of Hsp70 with radiofrequency ablation to increase tumor destruction by modulating Hsp70 production [27]. Therefore, it is necessary that therapeutic inhibition of Hsp70 be implemented to sensitize cancer cells toward heat stress and enhance tumor necrosis induced by hyperthermia. On the other hand, however, Hsp70 also serves as a danger signal which is closely related to the activation of immune system. Extracellular Hsp70 can induce chemokine production from tumor cells and are able to directly activate the chemo-attracted dendritic cells and macrophages through Toll-like receptor-4, resulting in the production of cytokines including IL-12, TNF-α and interferon [28–29]. However, to our knowledge, in the studies which used Hsp inhibitors to boost hyperthermia efficiency, few concerned the possible compromise of therapeutic effect caused by decreased immunogenicity resulted from inhibition of Hsp70.

Herein, AG was conjugated with IR820 for targeted tumor imaging and hyperthermia therapy. Cellular uptake and *in vivo* tumor imaging experiments were first performed to evaluate the targeting ability of AG-IR820. Then, the hyperthermia effect of AG-IR820 was evaluated *in vitro* followed by combined therapy at cellular level. Finally, the potent curative efficacy of the combined treatment was confirmed in nude mice bearing human thyroid duct carcinoma cells (TT).

## RESULTS AND DISCUSSION

### Synthesis and characterization of AG-IR820

**Figure 1:**
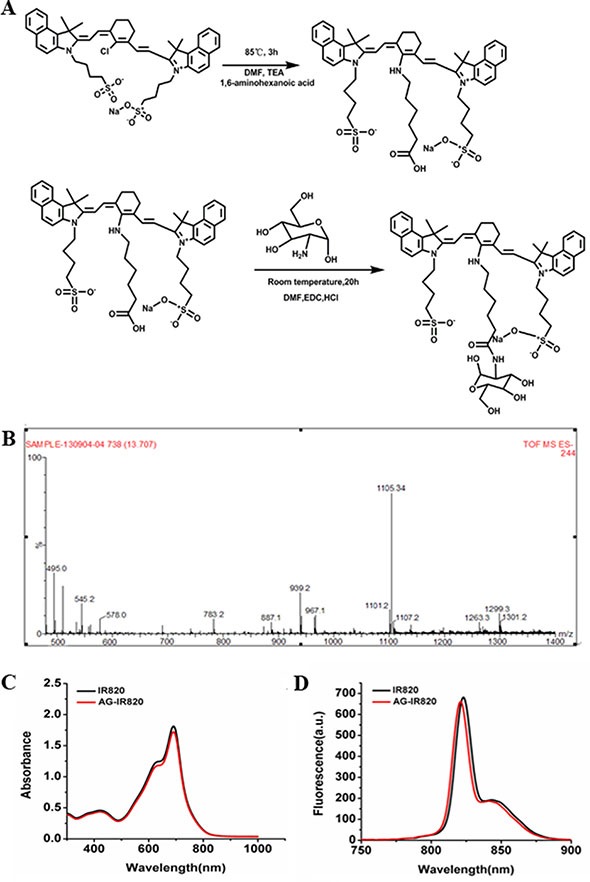
(**A**) synthetic scheme of AG-IR820; (**B**) mass spectrum of AG-IR820; (**C**) the absorption spectra of free IR820 and AG-IR820; (**D**) fluorescence emission spectra of free IR820 and AG-IR820.

In this study, one molecule of IR820 was conjugated to one molecule of AG. The covalent conjugation of AG-IR820 involved two steps as illustrated in Figure 1A. After purification, AG-IR820 was characterized by mass spectrometry. As shown in Figure 1B, the MS spectrum displayed that the molecular weight of AG-IR820 was 1105.34, consistent with the theatrical molecular weight of the final product in Figure 1A, indicating the successful preparation of AG-IR820. Then, we determined the fluorescence property of AG-IR820. As shown in Figure 1C, the absorption peak of both IR820 and AG-IR820 were at 691 nm. The emission spectra of IR820 and AG-IR820 were also identical, peaking at 823 nm (Figure 1D). These results proved that the conjunction of AG with IR820 didn't influence the fluorescence property of the latter.

### Tumor targeting ability of AG-IR820

#### Cellular uptake study

**Figure 2:**
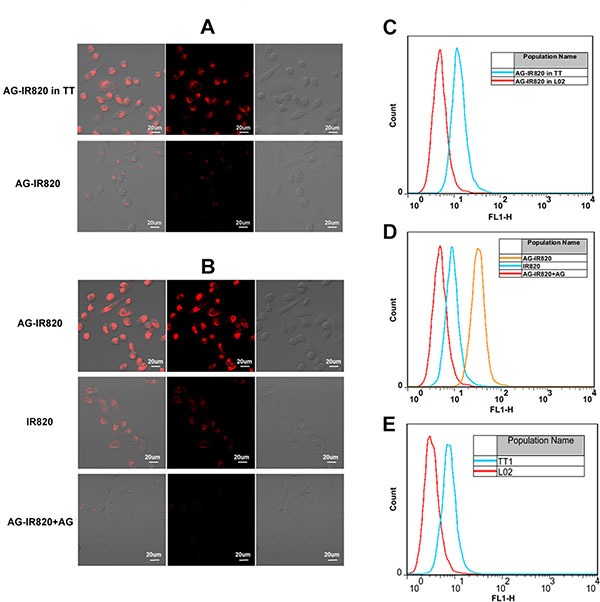
(**A**) Laser confocal microscopy image of TT cells and L02 cells treated with AG-IR820. (**B**) Laser confocal microscopy image of TT cells treated with AG-IR820, IR820, and AG block followed by AG-IR820. (**C**) Flow cytometry assay of TT cells and L02 cells treated with AG-IR820. (**D**) Flow cytometry assay of TT cells treated with AG-IR820, IR820, and AG block followed by AG-IR820. (**E**) GLUT1 expression comparison between TT cells and L02 cells by flow cytometry.

It has been well established that the glycolysis level and glucose consumption are much higher in malignant tumors [32]. As a result, it is postulated that the modification of IR820 with AG can facilitate greater uptake of the NIR dye by tumor cells. In this study, the cellular uptake ability of AG-IR820 and free IR820 in TT cells and uptake ability in L02 and TT cells were compared. As shown in Figure 2A, AG-IR820 in TT cells was significantly more than the probe in L02 cells. Cells incubated with AG-IR820 showed obviously higher fluorescence intensity than those treated with free IR820 (Figure 2B). To prove the role of AG in tumor specific uptake, the block experiment was performed by pre-incubating cells in the medium containing D-glucose before AG-IR820 treatment. The block group demonstrated drastically decreased fluorescence compared with the non-block group (Figure 2B). The flow cytometry assay was used as the quantitative method to compare the cellar uptake ability in each group. And the results were the same with confocal microscopy assay (Figure 2C and 2D). For explaining these results we also detected the GLUT1 expression in both TT and L02 with the flow cytometry. It showed us the expression of GLUT1 in TT cell line is obvious higher than in L02 cell line (Figure 2E). These observations confirmed the predominant role of AG modification in the enhanced cellular uptake of AG-IR820 probe, implying the potential of AG-IR820 for *in vivo* therapeutic studies.

### Tumor targeting ability *in vivo*

**Figure 3:**
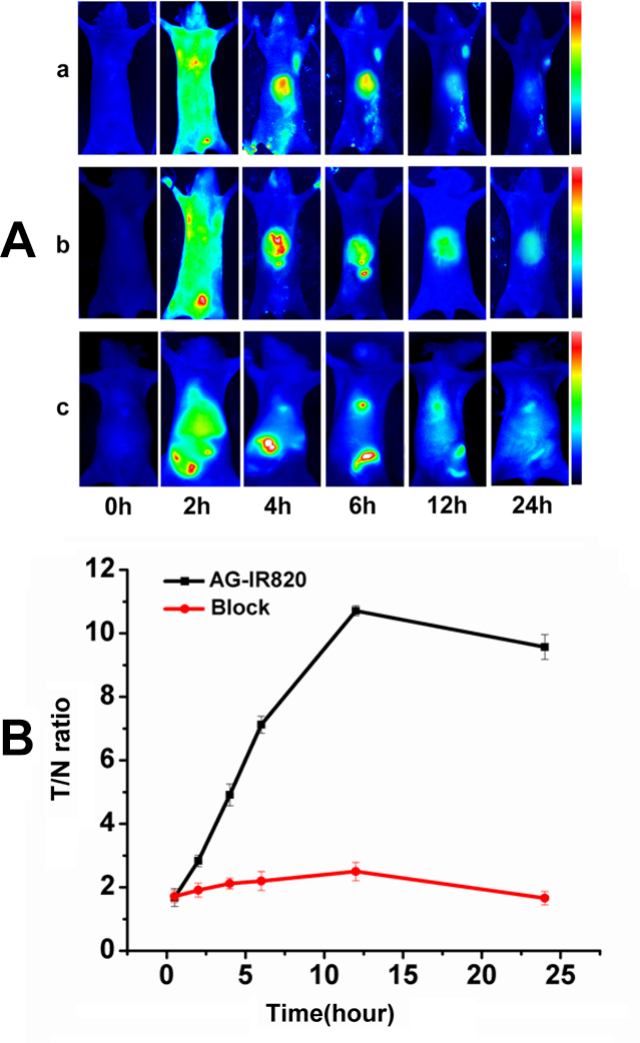
(**A**) Dynamics and tumor targeting ability of AG-IR820 in TT bearing nude mice (a), dynamics of AG-IR820 after AG block (b) and dynamics of free IR820(c); (**B**) Tumor/normal tissue ratio (T/N ratio = [tumor signal−background signal]/[normal signal (muscle)-background signal] × 100%) calculated from the ROIs at 2, 4, 6, 12, 24 hour postinjection of AG-IR820 and AG-IR820 with AG block.

The targeting ability of AG-IR820 was further evaluated in tumor bearing mice. As shown in Figure 3Aa, after AG-IR820 treatment, the fluorescence was quickly distributed all through the mice at about 2 h, with bright signal shown in tumor and liver at about 4 h. At 4 h post-injection, the tumor could be clearly distinguished from background tissues and tumor signal peaked at 12 h, which was still distinguishable at 24 h when the fluorescence signal was almost diminished in other parts including liver. To further verify the targeting ability of AG *in vivo*, we injected mice with free AG prior to AG-IR820 and the results showed that there was almost no fluorescence signal in the tumor site during 24 hours (Figure 3Ab). The free IR820 was injected in the tumor bearing mice and no tumor targeting ability was shown. The comparison of T/N ratio within 24 hours was demonstrated in Figure 3B. For the AG-IR820 group, the T/N ratio steadily increased from 0.5 h to 12 h and remained at a high level even after 24 hours. Contrastively, the T/N ratio in mice with AG pre-treatment was very low during the observation period. It could also be noted that the liver fluorescence in both groups increased during the first 6 hours and then gradually diminished afterwards. However, at 24 h, the liver signal in the block group was still distinguishable while the non-block group showed unobvious liver fluorescence. Because the concentration of the probe was the same for both groups, so the intensity of the fluorescence also kept the similar. In the non-block group some of the probe was concentrated in the tumor site, so the fluorescence intensity was lower than the block group in the liver. It was proved again that the AG agent could facilitate the IR820 retention in the tumor tissue. The preferred tumor accumulation of AG-IR820 and the prominent block effect of AG collectively proved that AG modification was the source of the evident tumor targeting ability. Moreover, AG-IR820 not only showed satisfying tumor targeting ability but is also observed with fast tumor accumulation for less than 4 hours, which is an advantageous property for *in vivo* imaging. The strong liver signal evidenced that the metabolism of AG-IR820 is mainly through liver. After the injection, the probe arrived at liver first and transferred from the bile duct to the intestine to have the enterohepatic secondary circulation. So we can see fluorescence in the liver. Importantly, the tumor retention time for AG-IR820 was admirable, with fluorescence signal still distinctive after 24 h. As the AG agent could make the probe stay in the tumor site for a long time, so it allowed for convenient image collection and efficient hyperthermia therapy. Compared with other NIR dyes with short plasma residence time, the relatively long *in vivo* circulation time of AG-IR820 allow for convenient image collection and efficient hyperthermia therapy.

### Cytotoxicity of IR820 and AG-IR820

**Figure 4:**
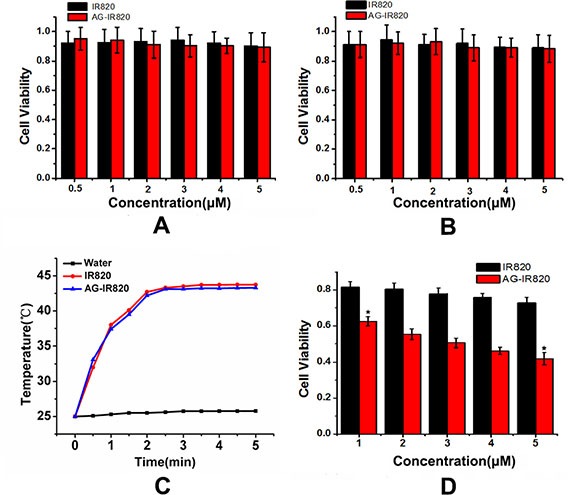
(**A**) Cytotoxicity of IR820 and AG-IR820 on TT cells; (**B**) Cytotoxicity of IR820 and AG-IR820 on L02 cells; (**C**) Temperature elevation of IR820 and AG-IR820 in aqueous solution; (**D**) *in vitro* photothermal ablation of TT cells by IR820 and AG-IR820. Data are given as mean ± SD (*n* = 5).

Before further application, we first determined the cytotoxicity of AG-IR820 using the methyl thiazolyl tetrazolium (MTT) assay. As shown in Figure 4A, the viability of TT cells was measured in the presence of IR820 and AG-IR820 at different concentrations from 0 to 5 μM. No obvious cytotoxicity was observed in both IR820 and AG-IR820 groups even at 5 μM. The same results were shown in AG-IR820 cytotoxicity assay in normal cells L02 (Figure 4B). The above results indicated that IR820 and AG-IR820 have good biocompatibility.

### Photothermal property of AG-IR820

The heat generation property of AG-IR820 was first determined in water solution. As shown in Figure 4C, temperatures of both IR820 and AG-IR820 solutions elevated to about 43°C at dye concentration of 5 μM, suggesting that the heat generating property of IR820 was not influenced by AG modification. Next, we evaluated the *in vitro* antitumor effect of hyperthermia induced by laser exposure on cells treated with IR820 and AG-IR820. Before NIR irradiation, cells were washed by PBS buffer for 3 times to remove the excessive dye not uptaken. As shown in Figure 4D, under laser exposure, AG-IR820 group demonstrated potent cytotoxicity effect in a dose department manner, with cell viability reducing to 42% at 5 μM dye concentration compared with the control group (*p* < 0.005). For the IR820 group, cell viability also decreased but to a much lower extent compared with that of the AG-IR820 group. Therefore, AG-IR820 is a more efficient hyperthermia agent to kill cancers than free IR820. As we washed the cells with PBS for 3 times, the photo-thermal effect was caused by the amount of dye inside cells. As a result, the improved hyperthermia efficacy was resulted from more dye uptake facilitated by AG modification. These observations again confirmed the important role of AG as a “warhead” to carry more desired agents into cancer cells and verified the heat generation property of AG-IR820 for hyperthermia therapy at cellular level.

### Combined treatment *in vitro*

**Figure 5:**
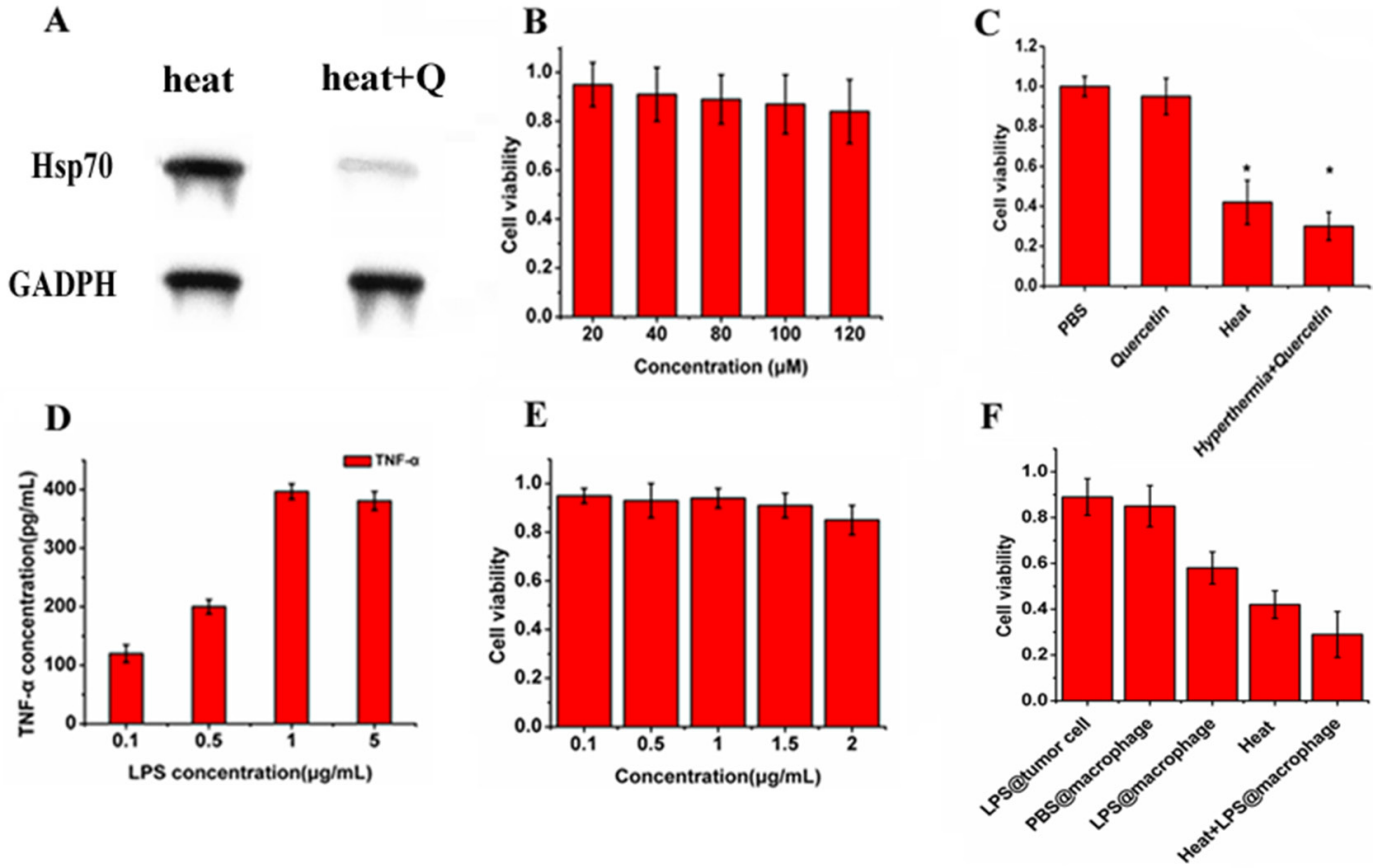
*In vitro* combined treatment with Quercetin and LPS (**A**) westernblot result of cells treated with heat stress and cells treated with Quercetin followed by heat stress; (**B**) cytotoxicity of Quercetin on TT cells; (**C**) effects of photothermal ablation of AG-IR820 combined with Quercetin pretreatment compared to PBS control, single Quercetin and single hyperthermia; (**D**) the release of TNF-α of macrophages induced by different concentrations of LPS; (**E**) cytotoxicity of LPS on TT cells; (**F**) viabilities of TT cells co-cultured with macrophages under different treatments (LPS directly on tumor cells, PBS on macrophages, LPS on macrophages, photothermal ablation combined with LPS on macrophages). Data are given as mean ± SD (*n* = 5).

To elucidate the combined effect of hyperthermia and Quercetin *in vitro*, we first evaluated Quercetin's ability to inhibit Hsp70 and its cytotoxicity. The westernblot results were shown in Figure 5A. For cells not pre-incubated with Quercetin, obvious Hsp70 expression was observed after heat treatment. However, pre-treatment of 100 μM Quercetin remarkably inhibited heat induced Hsp70 expression. In addition, cell viability was not evidently decreased by Quercetin even at 120 μM (Figure 5B). Therefore, Quercetin at 100 μM could effectively reduce the Hsp70 expression without obvious cytotoxicity. Then, we combined Quercetin with hyperthermia to confirm the improved cell inhibition efficiency caused by Hsp70 inhibition, where the photo-thermal effect was mediated by AG-IR820 treatment and laser exposure. Figure 5C showed that cell viability was evidently decreased in Quercetin + hyperthermia group compared with the group with hyperthermia alone. Given the high viability of cells treated with 100 μM Quercetin alone, it can be concluded that it is the inhibition of Hsp70 that synergize with hyperthermia to kill more cancer cells. In the figure 5C, we can see the cell viability in the heat group is about 40%, and the heat + Quercetin group could be about 28%. That is to say there is about 12% increase. Maybe it is not a very obvious, but after adding the Quercetin, the cell viability really decreased. The results told us it is worth to combine it to the photothermal with a fluorescent dye. Our result is consistent with previous studies using other cell lines to demonstrate the enhanced efficacy gained from such a combination [24, 33].

We also determined the *in vitro* effect of combined treatment with LPS stimulation. The detoxified LPS from E. coli serotype O55:B5 was used as the TLR-4 agonist in this study. The nature LPS also called endotoxin which has toxicity. Purified endotoxin is generally referred to as lipopolysaccharide or LPS, to distinguish it from the more natural completed cell membrane associated form. The core portion of the polysaccharide chain is common to LPS from wild and mutant bacterial strains. Removal of the fatty acid portions of lipid A results in detoxified LPS with an endotoxin level about 10000 times lower than that of the parent LPS. Figure 5D demonstrated the release of TNF-α by RAW264.7 macrophage cells stimulated by different concentrations of LPS. It was observed that the amount of TNF-α release increased with in LPS concentration in a dose dependent manner, which reached the peak at 1 μg/mL. Therefore, the macrophages could be effectively activated by LPS at 1 μg/mL. We then assessed the cytotoxicity of this type of LPS and found no obvious decrease of cell viability with LPS treatment up to 2 μg/mL (Figure 5E). This result confirmed that the LPS used in this study induced no obvious cytotoxicity, showing a favorable biocompatibility. To further illustrate the anti-proliferation effect of activated immune cells on tumor cells, we co-cultured RAW264.7 cells with TT cells in transwell plates. As shown in Figure 5F, the cell survival rate was drastically decreased if LPS was added to macrophages in the upper chamber. When hyperthermia was given, the co-cultured tumor cells lost more viability. Therefore, O55:B5, a purified and detoxified LPS, may be effective *in vivo* to wake immune cells up and kill cancer cells and contribute to improve the therapeutic effect of hyperthermia. Overall, the *in vitro* efficiency of such combinations laid the foundation for further *in vivo* application.

### Antitumor efficacy *in vivo*

**Figure 6:**
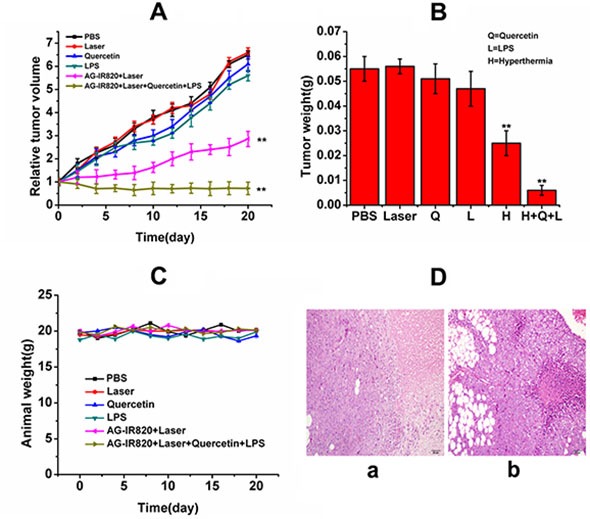
*In vivo* antitumor efficacy of different treatments on TT tumor bearing nude mice (**A**) relative tumor volume of mice treated with PBS, laser, Quercetin, LPS, AG-IR820 + laser, AG-IR820 + laser + Quercetin + LPS; (**B**) tumor weights of TT tumor bearing mice under different treatments; (**C**) weights of mice bearing TT tumor under different treatments during 20 days treatment. Data are given as mean ± SD (*n* = 6). **P < 0.01. (**D**) hematoxylin and eosin-stained tumors saline treated mice (a) and AG-IR820 + laser + Quercetin +LPS treated mice (b).

Although satisfying *in vitro* effects of hyperthermia using AG-IR820 have been achieved, the *in vivo* efficacy may be complicated by various factors. Some the key limitations of hyperthermia treatment include the difficulty to realize targeted delivery of heat generating agents and insufficient ablation by hyperthermia alone. [34]. Therefore, we combined other therapeutic modalities with hyperthermia, including Hsp70 inhibition and tumor immunogenicity enhancement to strengthen the therapeutic effect of heat ablation. In the TT tumor model, the initial tumor volume at day 0 was normalized to 1, which was used to calibrate the average tumor volumes at different days of treatment as shown in Figure 6A. It was found that compared with the control group, mice treated with Quercetin showed almost no difference in tumor growth, with relative tumor volume above 6 at 20 day treatment. The LPS treatment caused slightly reduced tumor volume but showed no significant difference compared with the control group. Although unobvious, the improved antitumor efficacy of LPS treatment may be caused by the activation of innate immune response in tumor tissues with subsequent recruitment of cytokines such as TNF-α and IL-6. Nonetheless, these agents alone couldn't effectively induce tumor necrosis without hyperthermia. For mice treated with hyperthermia alone, obvious tumor inhibition was observed and the relative tumor volume was reduced to 2.8 due to heat ablation of tumor cells. The most remarkable tumor shrinkage was achieved in the triple treatment group, with relative tumor volume of 0.72 at the end of treatment. Therefore, the final combined treatment integrated the benefit of each single agent and resulted in an impressive antitumor efficacy. The data of tumor weight was consistent with tumor volume. As shown in Figure 6B, the tumor weight in the triple treatment group drastically decreased to 0.008 g compared to 0.07 g in the control group at day 20. Moreover, no obvious weight loss was found in mice of each group (Figure 6C), indicating low systematic toxicity of our treatment regimen in spite of pronounced tumor inhibition. The tumor inhibition rates were also calculated as 6.15% (Quercetin), 13.8% (LPS), 56% (Hyperthermia alone) and 89% (Quercetin + hyperthermia + LPS) respectively, which again confirmed the integration of the three therapeutic modalities as a novel and effective strategy to treat thyroid cancer. After the administration, the tumor of saline and combined treatment group were collected for histological examination. pronounced pathological changes were revealed (Figure 6D).

In this study, we used LPS as a compensatory therapeutic agent because Hsp70 was inhibited, which represents a well characterized endogenous danger signal igniting immune response after combining TLR-4 on DC cells or macrophages, pointing to the need of an exogenous agonist. The further enhanced antitumor efficacy of our three-punch therapy is most likely due to the activation of innate immune response by LPS and subsequent release of antitumor cytokines through TLR-4 pathway. Similar design was used in a recent study [35], where Dendrophilin, another kind of purified LPS was used to compensate the compromised immunogenicity of HMGB1-deficient tumors in chemotherapy. In hyperthermia therapy, however, there is a contradiction as whether the benefit of Hsp70 inhibition can outweigh its effect on immune activation. Taoyong Chen et al. [28] have provided detailed evidence that Hsp70 released from heat-stressed tumor cells can initiate antitumor immunity by activating dendritic cells via TLR-4 pathway, and many other studies also demonstrated similar conclusion, suggesting the benefits of immune stimulation effect of Hsp70 in hyperthermia treatment. On the other hand, as we have introduced above, the inhibition of Hsp70 does result in a pronounced heat ablation effect. It is possible that hyperthermia alone cannot provide enough stimulation to reverse the immunosuppressive condition in the complex tumor environment *in vivo*, which fails to counter the cyto-protective effect caused by the boosted Hsp70 expression. Even though we cannot give a final explanation, it is obvious that inhibition of Hsp70 would bring a better therapeutic result, and with further combination with LPS treatment, a much optimized therapeutic outcome can be achieved.

Nonetheless, the synergistic mechanism of the combination of Hsp70 inhibitor and TLR-4 agonist to promote hyperthermia therapy needs to be further characterized. For example, the extent of the compromise of immune response after Hsp70 inhibition should be elucidated, and how LPS treatment restores immunogenicity is required to be monitored both *in vitro* and *in vivo*. These issues will be the focus of our future study.

## MATERIALS AND METHODS

### Reagents, cell culture and instrumentation

Amino-glucose (85%), IR820, methanol, dimethylsulfoxide and 6-aminohexanoic acid, dimethylformamide were purchased from Sigma–Aldrich. Human thyroid cancer cell line TT was purchased from Cell Bank of Chinese Academy of Sciences (Shanghai). F-12K culture medium, fetal bovine serum (FBS) were purchased from Life technologies. Dulbecco's Phosphate Buffered Saline (DPBS) was purchased from Sigma Aldrich. Abortion measurements were performed using UV– vis spectrophotometer (JH 754PC, Shanghai, China). The high-performance liquid chromatography (HPLC) system (Waters) was used to purify products. Product identification was completed by Q-TOF Micro Mass Spectrometer (Waters). Laser confocal microscopy (Olympus) was used to obtain *in vitro* fluorescence signal in cells. Fluorescence signal of samples was acquired by NIR spectral system. The home-made NIR imaging system was used for real time detection of fluorescent signal in mice. Briefly, the NIR system contains an excitation laser (λ = 765.9 nm, NL-FC-2.0-763 laser light), a high sensitivity NIR CCD camera (PIXIS 512B, Princeton Instrumentation) and an 800 nm long pass filter for capturing the fluorescence emission from the tissue. In addition, another HLU32F400 808 nm laser (LIMO, Dortmund, Germany) was incorporated as background light to obtain the animal profile.

### Preparation and characterization of AG-IR820 conjugate

The synthesis of AG-IR820 conjugate was similar to that of chitosan-IR820 reported by Masotti et al. [34]. First, the intermediate product IR820 linker was prepared with meso-chlorine group of IR820 substituted by 6-amino hexanoic acid, introducing a carboxyl end for further modification. Specifically, 0.3 mmol IR820 was reacted with 1.5 mmol 6-aminohexanoic acid acid and 1.42 mmol triethylamine under magnetic stiring in 1.2 ml anhydrous DMF. The reaction solution was purged with Nitrogen gas for 20 minutes. Then, a 3 h stirring at 400 rpm was performed for the resulting green solution in a corked flask with oil bath to maintain a constant temperature of 85°C. The final solution was obtained after the color turned blue followed by freeze-drying and lyophilization, which gave a yield of 70%. The next step before the final conjugation with AG involved the functionalization of the IR820 linker to form an ester by N-(3-dimethylaminopropyl)-N-ethylcarbodiimide hydrochloride (EDC), a zero-length cross-linker. Then, the fictionalized IR820 linker was added with 2.5 ml AG drop-wise and the reaction lasted for 24 h, forming an amide bond between AG and the ester linker. The final product presented a yield of 60%. Then, dialysis was performed for purification with a cut off membrane of 100-300 kDa against the mixture of methanol and water (methanol: water = 70%:30%) and then with 100% water for 3 days followed by the final freeze-drying for recovering. The solutions of IR820 and AG-IR820 conjugate were prepared in deionized water. The samples were then dilluted serially to a linear range and measured immediately after preparation. The absorption and fluorescence properties were recorded on the UV-vis spectrophotometer and an NIR spectral system respectively. The identity of the compound was confirmed by Q-TOF micro mass spectrometer (MS) and NMR spectrum.

### Preparation of Quercetin-loaded liposomes

Quercetin-loaded liposomes were prepared according to previous report by Doxil [35]. Briefly, in chloroform, the mixtures of hydrogenated soy phosphatidylcholine/cholesterol/polyethyleneglycol phosphatidylethanolamine (PEG2000-PE) were prepared with mol ratio of 57.25:37.57:5.18, which were followed by addition of 0.25 mg of Quercetin. After the lipid film was formed by solvent removal, rehydration was performed and the preparation was probe-sonicated using sonic dismembrator (Fisher Scientific) at 7W for 30 min. The sample was then centrifuged for 10 min at 2000 rpm. 0.35 mg of Quercetin was loaded in each administrated dose. The size and Zeta potential of the liposome formulation was 120 ± 35 nm and – 37.1 ± 5.5 mV, respectively. The TEM figure was shown in Supplementary Figure 1.

### Tumor targeting assay of AG-IR820

### Cellular uptake study

In order to explore different cellular uptakes between IR820 and AG-IR820 conjugate and the cellular uptakes of AG-IR820 between human normal liver cells L02 and TT cells, cellular uptake and imaging experiments were performed. The flow cytometry was used for the quantity assay. Briefly, TT cells were routinely cultured in F-12K medium and incubated with 5% CO_2_ at 37°C and seeded in two 15 mm glass-bottomed cell culture dishes. After attachment over 12 hours, culture medium was replaced by the medium containing 5 μM IR820 or AG-IR820. The same way with the L02 and TT cells incubated with AG-IR820. Then, the wells were kept for 5 hours in dark followed by 3 times washing with Phosphate Buffered Saline (PBS) and subjected to laser confocal microscopy. The competitive effect of glucose was tested by pre-incubating cells with 50 mM D-glucose for 30 min at 37°C followed by adding 5 μM AG-IR820. After PBS washing, cells were observed under laser confocal microscopy. The cells of L02 and TT were treated with the same way after incubated with AG-IR820 were detected by flow cytometry. The expression of GLUT1 between L02 and TT was studied by flow cytometry.

### *In vivo* tumor targeting assay

Athymic nu/nu nude mice of 4–6 weeks old were purchased from Charles River Laboratories (Shanghai, China). All animal protocols were approved by the guidelines for animal use of Jilin University. The nude mice were subcutaneously injected with approximately 2 × 10^6^ cancer cells in 0.2 ml phosphate buffered saline (PBS) to axillary fossa for each. After tumor volume reached about 0.5 cm in diameter, the nude mice were randomly divided into 2 groups with 6 mice in each. Afterwards, mice in group 1 were injected with AG-IR820 at dye dose of 10 mg/kg via tail vein. To further confirm the effect of AG, mice in group 2 were pre-injected with D-glucose 2 hours before AG-IR820 treatment. Images were collected at time points of 2 h, 4 h, 6 h, 12 h, and 24 h after dye injection, which was followed by calculation and comparison of tumor/normal tissues ratio (T/N ratio). T/N ratio was obtained by ROI function as T/N ratio= (tumor signal-background signal)/ [normal signal (muscle)-background signal]. Data are expressed as a means SD (*n* = 6).

### Cytotoxicity assay

MTT assay was performed to determine the cytotoxicity of IR820 and AG-IR820 conjugate. Cells were seeded in 96-well plate and incubated for 12 h to allow for confluence. After sufficient attachment, the cells were treated with free IR820 and AG-IR820 at different IR820 concentrations ranging from 0 to 5 μM. After 24 h incubation, cell viabilities were evaluated through MTT assay.

### Photothermal evaluation of IR820 and AG-IR820

To determine hyperthermia effect on cell growth, the photo-thermal effects of AG-IR820 and IR820 were first characterized in PBS solution. IR820 and AG-IR820 solutions (1 mL) of dye concentration of 5 μg/mL were irradiated at 808nm wavelength (8 W/cm^2^) and the temperature changes were monitored simultaneously in 5 minutes. To evaluate the *in vitro* hyperthermia ablation effects, TT cells were seeded and incubated with IR820 and AG-IR820 with dye content of 5 μM for 1 h followed by washing with PBS for 3 times. Then, the cells were exposed to 808nm NIR light for 5 minutes at 8.8 W/cm^2^. After laser exposure, cells were incubated again to reach 24 h incubation time, and MTT assay was performed to determine cell viability. In each test, 5 parallel wells were set and averaged to obtain the cell viability data which were normalized to that of control group and represented by mean ± S.D. The statistical difference among different groups was analyzed by One-way ANOVA, and significance was set at *p* < 0.05.

### Effects evaluation of Quercetin and LPS

Western blot assay was used to test the inhibition of Hsp70 by Quercetin. 2 groups of TT cells were subjected to sublethal heat stress of 40°C for 35 min, for which group 1 was pre-incubated with 100 μM Quercetin 2 h before laser exposure. Westernblot assay was performed 4 hours after heat stress. Briefly, cells were first lysed by lysis buffer and the resultant cell extracts were centrifuged for 10 min at 10,000 g. Then, the supernatant was collected and boiled in Laemmli sample buffer and subjected to 10% poly-acrylamide gel electrophoresis. The separated proteins were electrophoretically transferred to a polyvinylidene fluoride membrane and blocked for 1 h and probed using rabbit anti-Hsp70 Ab (1:1,000 dilution, Cell Signaling Technology) and rabbit anti-β-actin Ab (1:1,000 dilution, Cell Signaling Technology). The blotts were then washed 3 times with 0.2% Tween-20-TBS, and incubated for 1 h with horseradish peroxidase-conjugated donkey anti-rabbit IgG (1:10,000 dilution, Cell Signaling Technology). After washed three times with 0.2% Tween-20-TBS again, the blots were visualized using ECL (Millipore) and exposed to X-ray film. Elisa assay was used to quantify the release of TNF-α of macrophages after LPS stimulation. Anti-mouse TNF-α and biotin conjugated anti-mouse TNF-a cocktail were purchased from Cell Signaling Technology. The mouse leukemic monocyte macrophage cell line (RAW264.7 cell line) was purchased from Cell Bank of Chinese Academy of Sciences (Shanghai). Detoxified LPS from E. coli serotype O55:B5 was obtained from Sigma-Aldrich. Cells were isolated and suspended in DMEM medium and then cultured in 6 well plates with the density of 3 × 10^6^ cells per well. Afterwards, the cells were treated with LPS of different concentrations (0.1, 0.5, 1 and 5 μg/ mL) respectively. The supernatant of cell culture were harvested after 3 hours and TNF-α were determined using corresponding ELISA kit.

### Hyperthermia with Quercetin

For combined treatment with Quercetin, cells were first incubated with both 5 μM AG-IR820 and 100 μM Quercetin 2 hours before laser exposure and then subjected to NIR light at 808 nm wavelength for 5 min. After 24 h incubation, cell viabilities were evaluated through MTT assay.

### Hyperthermia with LPS stimulation

Transwell plates were used to test the inhibition of tumor cells co cultured with macrophages which were treated by LPS. Briefly, RAW264.7 cells (1.5 × 10^5^ cells/ mL) and TT cells (5 × 10^4^ cells/ml) were incubated in the upper and lower chambers respectively. LPS and PBS solution were added to different upper chambers. For combined treatment, macrophages in the upper chamber were incubated with LPS and TT cells in the lower chamber were treated with AG-IR820. At 1 h after incubation, cells in lower chambers were exposed to NIR laser at 808nm wavelength for 5 minutes (8 W/cm^2^). After the total 24 h incubation was completed, MTT solution was added to the lower chambers for 4 h incubation. Cell viability was then determined with 5 replicates of each treatment, and the results were normalized to the control group.

### Combined therapy *in vivo*

Nude mice were inoculated with tumor cells as described above and randomly assigned into 6 groups after tumor size reached about 0.5 cm in diameter with 6 mice in each group. Mice were treated with single therapy or combined treatment. Specifically, mice in group 1 and 2 were only treated with PBS and laser (808 nm, 8 W/ cm^2^, 5 min) respectively as control. Mice in group 3 and 4 were treated with Quercetin (0.20 mg in 0.2 mL) and LPS (3 μg in 0.1 mL 0.9% NaCl) as single therapy respectively. For hyperthermia, mice in group 5 were injected with AG-IR820 (15 mg/kg) followed by laser exposure (808 nm, 8 W/cm^2^, 5 min). For the combined therapy, mice in group 6 were first injected with Quercetin (0.20 mg in 0.2 mL) and then, 12 hours later, AG-IR820 was injected, which was followed by laser treatment 24 hours after Quercetin injection. (There is no strict limitation to the imaging probe concentration for the tumor detection *in vivo*. So in the *in vivo* imaging study we tried with several different doses of AG-IR820 and chose 10 mg/kg at last because the better imaging property it showed. In the *in vivo* therapeutic study, we increased the dosage of the probe to 15 mg/kg for the hypothermia treatment and got the good effect.) Finally, LPS (0.2 mg in 0.2 mL) was administrated at 12 h after laser ablation. Laser treatment was repeated for 3 times at day 3, 6 and 9 respectively. To monitor tumor development, the tumor volume was determined at 2 days interval according to the equation: V = L ×W^2^/2, where W and L indicate tumor width and tumor length measured at the widest and longest points respectively. Mice weight was determined every the other day during the 20 day treatment. At the last day of treatment, the mice were sacrificed and tumor weight and tumor inhibition rates were calculated.

## CONCLUSION

In conclusion, we have rationally developed a novel therapeutic strategy for thyroid cancer treatment which took advantages of targeted hyperthermia, heat sensitization by Hsp70 inhibitor Quercetin and enhanced immunogenicity by LPS. AG-IR820 probe was successfully constructed for *in vivo* tumor imaging and hyperthermia therapy. LPS treatment served to compensate the loss of immunogenicity caused by Hsp70 inhibition and thus provided a new approach to perfect the “Hyperthermia + Hsp70 inhibitor” mode which has already been widely reported. These modalities collectively contributed to the optimized therapeutic outcome both *in vitro in vivo*, which was confirmed in the treatment of tumor bearing nude mice. More importantly, given the limitations of surgery and radiotherapy, our study enriched the arsenal of therapeutic weapons for combating thyroid cancer.

## SUPPLEMENTARY MATERIALS FIGURES


