# Supplementary material for: Targeted near infrared hyperthermia combined with immune stimulation for optimized therapeutic efficacy in thyroid cancer treatment
Source: Oncotarget. 2016 Jan 12;7(6):6878–90. doi: 10.18632/oncotarget.6901 (PMC4872755; doi:10.18632/oncotarget.6901)
Supplement: Supplementary file 1 [file oncotarget-07-6878-s001.pdf]

Targeted near infrared hyperthermia combined with immune stimulation for optimized therapeutic efficacy in thyroid cancer treatment

Supplementary Materials

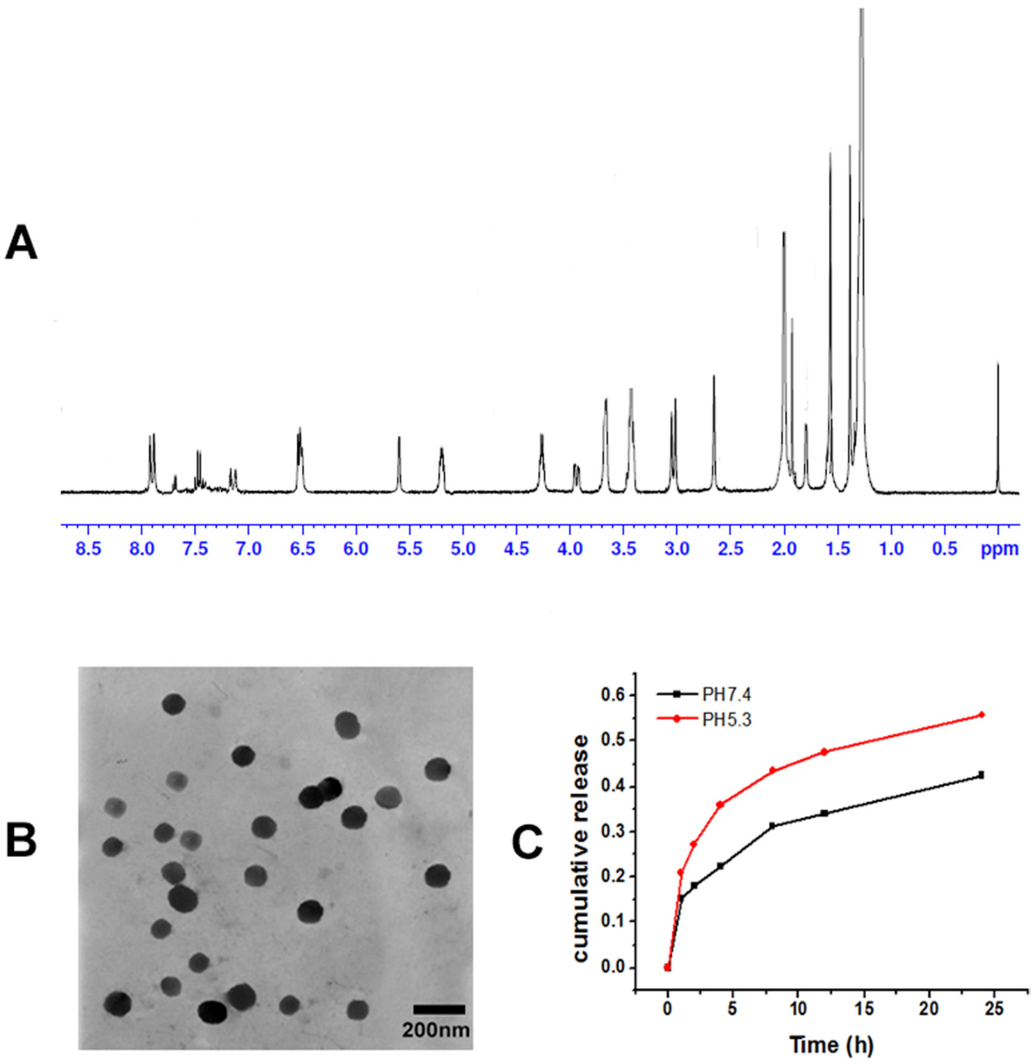

Supplementary Figure S1: (**A**) NMR spectrum of the probe AG-IR820; (**B**) TEM picture of the Quercetin-loaded liposomes; (**C**) Release rate of the Quercetin-loaded liposomes.

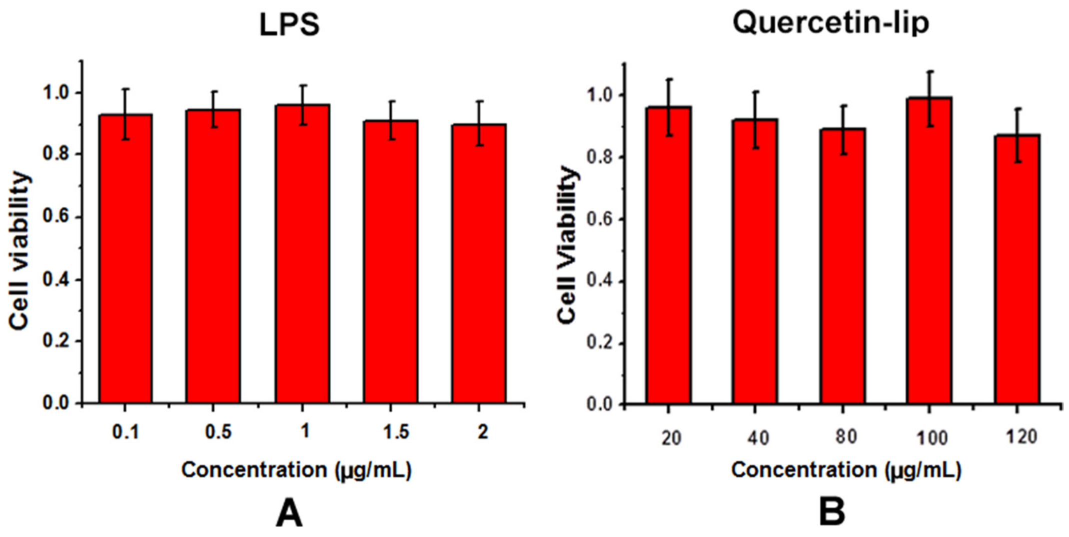

Supplementary Figure S2: (**A**) cytotoxicity of LPS on human normal liver cells L02. (**B**) cytotoxicity of Quercetin-lip on human normal liver cells L02.

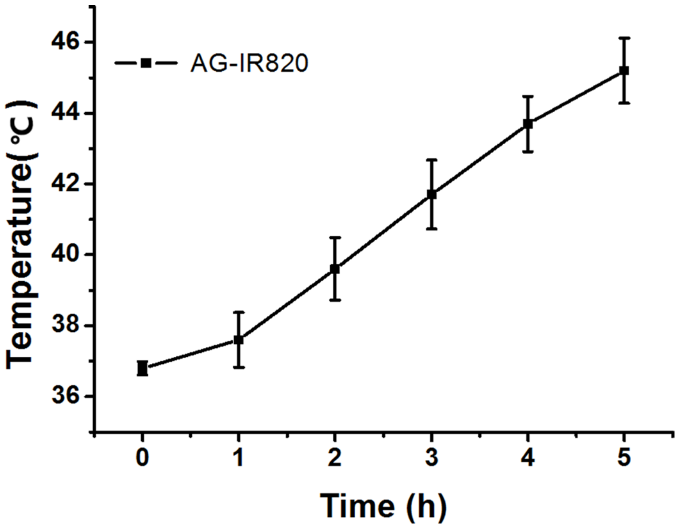

Supplementary Figure S3: Temperature elevation of AG-IR820 in the tumor tissue.

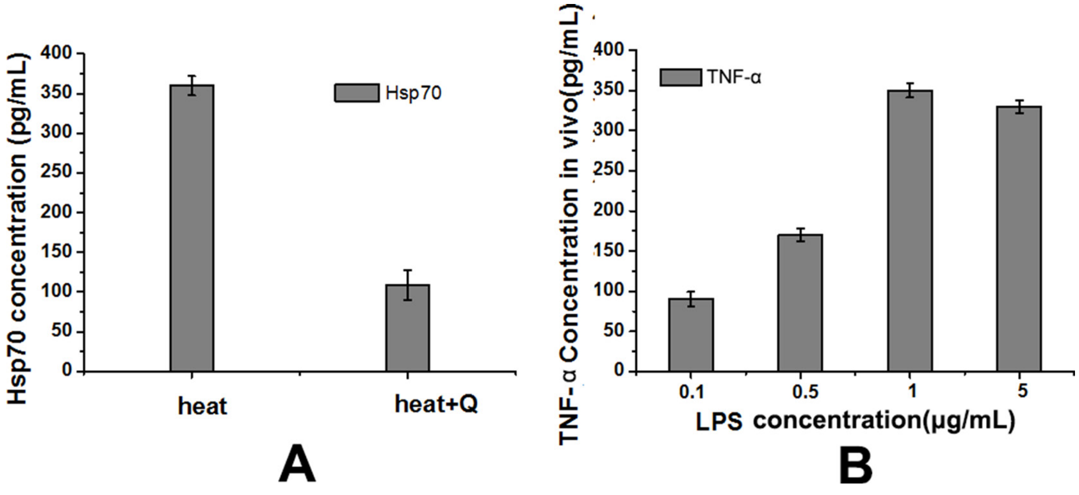

Supplementary Figure S4: (**A**) Hsp70 expression in tumor tissue comparison between with or without Quercetin-lip injected by Elisa method. (**B**) TNF-α expression in tumor tissue comparison between with or without LPS injected by Elisa method.
